# Enterobius Vermicularis: A Parasitic Cause of Appendicular Colic

**DOI:** 10.7759/cureus.8524

**Published:** 2020-06-09

**Authors:** Abhishek Chitnis, David Yousefi Azimi, Shariq Sabri, Alhad Dhebri

**Affiliations:** 1 General Surgery, Tameside General Hospital, Manchester, GBR

**Keywords:** enterobius vermicularis, parasite, pinworm, appendicular colic, acute appendicitis, appendicectomy, appendicitis

## Abstract

*Enterobius vermicularis* is the most commonly identified parasite incidentally found within the appendix of a clinically diagnosed appendicitis. This parasitic cause of appendicular colic, primarily affecting children, is an important cause of negative appendicectomy. We report an unusual and interesting case of a young female who presented with clinical features of acute appendicitis. Laparoscopic appendicectomy revealed the presence of an *Enterobius vermicularis *infestation originating from the lumen of her vermiform appendix. Our case report is supplemented with a review of the literature, an overview of the parasitology, and discussion of pertinent symptomatology and peri-operative management strategies.

## Introduction

The nematode *Enterobius vermicularis*, widely known as pinworm, is the foremost parasitic cause of gastrointestinal infection worldwide [1-4]. It is also the most commonly identified parasite incidentally found within the appendix of a clinically diagnosed appendicitis [2-5].

The worldwide association of *Enterobius vermicularis* infestation with acute appendicitis ranges extensively from 0.2% to 41.8%, with an overall average global prevalence of 4% [1,5,6]. Although *Enterobius vermicularis* infection can affect patients of all ages, it primarily resides in children, affecting between 4% and 28% of children worldwide [1-3].

Transmission is most common via the faecal-oral route and following ingestion of infected eggs, the larvae hatch in the stomach and small intestine. They then travel to the colon, mainly to the caecum and appendix, and mature into adult pinworms [1,3,5-7].

Parasites can cause appendicitis by obstructing the lumen of the appendix or by causing secondary inflammation. However, throughout the literature, histopathological examination of appendicectomies has demonstrated a large proportion of cases causing no acute inflammation in the appendiceal wall, thus an absence of acute appendicitis, and a cause of a negative appendicectomy [1-4,6,8].

We report here our experience of a young female who presented with clinical features of acute appendicitis, but was found to have an *Enterobius vermicularis* infestation of her vermiform appendix during laparoscopic appendicectomy. We also review the published literature of this parasitic cause of appendicular colic to highlight the epidemiology, common presenting features, diagnosis, investigations, and pharmacological and peri-operative management strategies.

## Case presentation

A 17-year-old Caucasian female was admitted to the paediatric department with a two-day history of right iliac fossa abdominal pain associated with pruritus ani, nausea and anorexia. She reported no change in her bowel habits, weight loss or urinary symptoms.

On admission, her physiological parameters revealed a low-grade pyrexia of 37.7°C but otherwise normal observations, including a heart rate of 80 beats/minute, a blood pressure of 111/66 mmHg, a respiratory rate of 18 breaths/minute and an oxygen saturation of 98% on room air. Physical examination elicited severe tenderness on palpation of the right iliac fossa, with a focus at McBurney's point and a positive Rovsing's sign. Unfortunately, digital rectal or pelvic examinations were not performed by the clerking clinician.

Laboratory tests revealed an elevated white cell count and C-reactive protein. Other investigations, including urinalysis, pregnancy test and abdominal ultrasound, were all unremarkable.

**Figure 1:**
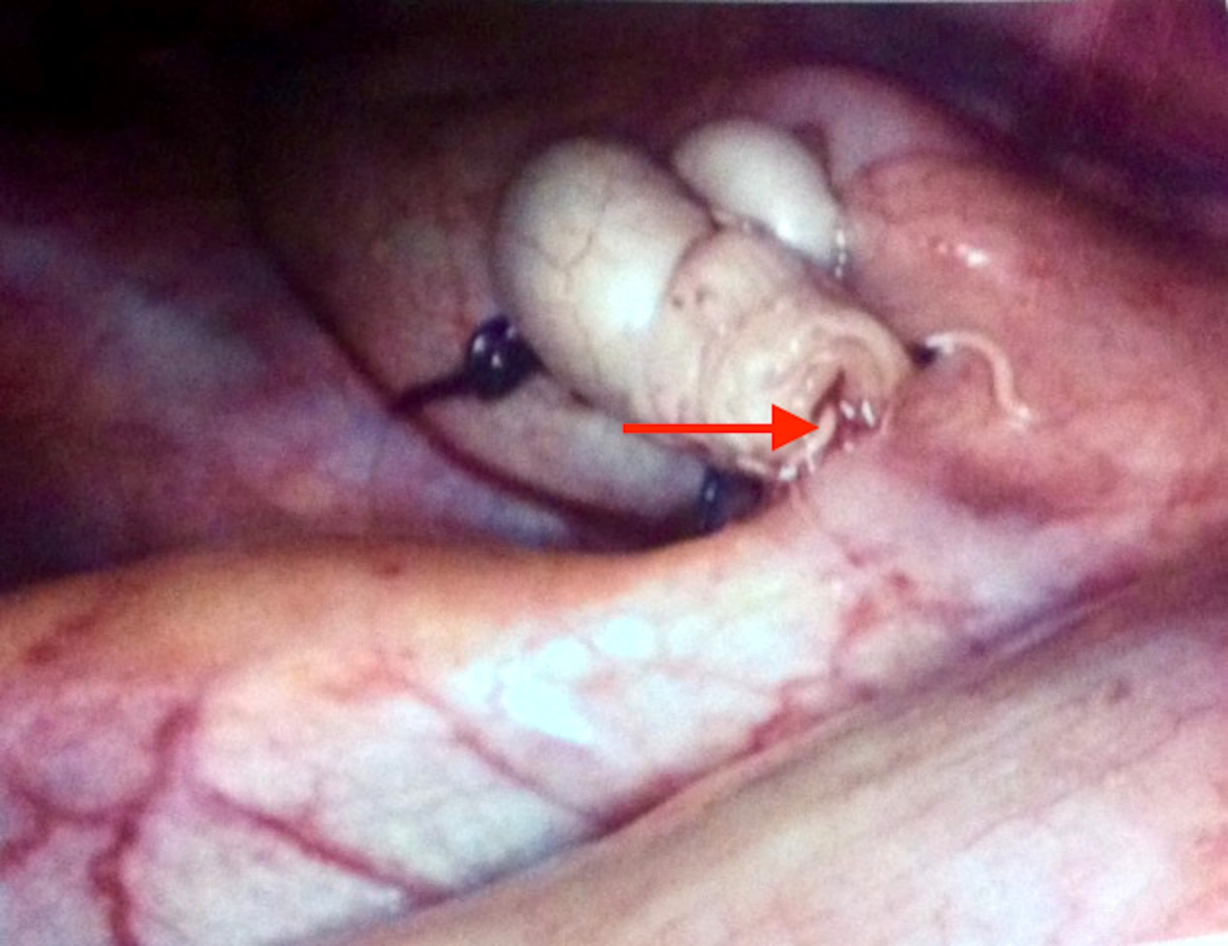
Enterobius vermicularis infestation in the lumen of a vermiform appendix (red arrow)

The general surgeons were consulted for a clinical review of suspected acute appendicitis. Subsequent laparoscopic appendicectomy revealed an incidental finding of macroscopically visible *Enterobius vermicularis *originating from the lumen of a lily-white appendix (Figure 1). The histopathological report revealed no inflammatory infiltration of the underlying appendiceal mucosa.

The patient's postoperative recovery was uneventful. Both the patient and her family members (who were thought to be *Enterobius vermicularis* carriers) received one oral dose of mebendazole 100 mg that was repeated after two weeks, as per hospital and British National Formulary guidelines. The follow-up to date was without incident, and the patient was free of symptoms one year after the operation.

## Discussion

The global burden of *Enterobius vermicularis* in appendicitis is reflected in the meta-analysis by Taghipour et al. [5]. This review demonstrated that out of 103,195 cases of appendicitis, 3,005 (2.9%) were secondary to histologically confirmed *Enterobius vermicularis* infection. The meta-analysis also revealed a geographical variation ranging from 2% incidence in the Americas to 8% in Africa, which was attributed to differences in lifestyle, sanitation status, socioeconomic conditions and climate [5].

It is important to note that although the prevalence of *Enterobius vermicularis* has significantly decreased in the recent decades, in part secondary to screening programmes and improved public health services, most infected subjects are asymptomatic and may be misdiagnosed [5].

An important red flag symptom that infected patients commonly present with is perianal pruritus, especially at night. This is due to the life cycle of *Enterobius vermicularis*, in which gravid females migrate nocturnally outside of the anus and oviposit while crawling on the skin of the perianal region [1,3,7,9]. This is further linked to the behaviours of young patients, such as finger sucking and anal scratching, which may also lead to excoriations and bacterial superinfection [3,7,9].

Other signs and symptoms of *Enterobius vermicularis* may mimic acute appendicitis due to parasites obstructing the lumen of an appendix, thereby causing appendiceal colic [1-4,6,8]. Moreover, *Enterobius vermicularis* infections can also cause chronic abdominal pain, urinary tract infections, eosinophilic ileocolitis and pelvic abscesses [1,4-6]. Other pathologic changes ranging from lymphoid hyperplasia to acute phlegmonous inflammation with life-threatening complications, such as gangrene and peritonitis, have also been reported [2-4,8,9].

Epidemiologically, the prevalence of *Enterobius vermicularis* is significantly higher in females than in males, and more common in girls with the average age of patients being 12 years [5,6]. Therefore, it is important to consider *Enterobius vermicularis* in female gender-specific pathologies, particularly female genital tract infections, such as vulvitis, vaginitis and salpingitis [5-7].

As demonstrated in the literature and from our case report, there is a high proportion of negative appendicectomies performed on patients with concurrent clinically diagnosed appendicitis and *Enterobius vermicularis* infestation. Ilhan et al. observed a negative appendicectomy rate of 52.7%, in which there were no findings of acute appendicitis [4]. The authors also cite six studies that report rates between 25% and 85.7% of no acute inflammation within appendiceal specimens [4].

It therefore seems pertinent that attempts should be made to incorporate a routine screen of appendicitis in higher risk cases of *Enterobius vermicularis*, to confirm a diagnosis prior to surgery [5]. Diagnostic methods include observation of worms in the perianal region two to three hours after sleeping or analysing samples from under fingernails for microscopy [7]. Diagnosis can also be made using saline swabs or adhesive tape to collect *Enterobius vermicularis* eggs from the perianal skin in the early morning [5-7,9]. These tests have however been shown to have a low sensitivity because of the variable emergence of worms at the anal verge, and the intermittent nature of egg laying with respect to the parasitic life cycle [9]. If performed, a digital rectal and pelvic examination in our case may have nevertheless elucidated a diagnosis of a parasitic infection.

Additional diagnostic investigations to perform include laboratory tests and ultrasound. Evidence of normal laboratory tests, including white cell count, neutrophil count and C-reactive protein, at presentation helps to predict *Enterobius vermicularis* infection in children who present with right iliac fossa abdominal pain [3]. Other studies have also shown the white cell count in *Enterobius vermicularis* positive patients to be significantly lower than that of *Enterobius vermicularis* negative patients [3].

The use of ultrasound for right iliac fossa abdominal pain is also a useful diagnostic imaging technique for evaluation of acute appendicitis, and is superior to laboratory tests for both confirming and excluding appendicitis [3]. However, ultrasound was shown to not be a reliable method to diagnose *Enterobius vermicularis* infestation of the appendix, with almost half (48.7%) of *Enterobius vermicularis* positive patients having a negative ultrasound result [3]. The results of our case report correlate with the above evidence.

An over-reliance on CT scanning has been reported for the evaluation of clinically suspected appendicitis in children [10]. However, guidelines focussing on early surgical consultation for high clinical suspicion of acute appendicitis and an ultrasound scan for patients with equivocal or non-classical clinical findings demonstrated a marked decrease in CT use for patients undergoing appendicectomy without an increase in the negative appendicectomy rate [10]. In addition, there are concerns about the risks of malignancy associated with CT radiation, which are greater in paediatric patients due to increased radiosensitivity and more years of life during which radiation-induced malignancy could develop [10]. CT scanning is therefore usually reserved for patients with a high clinical suspicion for perforated appendicitis or if ultrasound is unavailable, which was not applicable to our case.

Currently, there is no consensus on whether a macroscopically normal appendix found during laparoscopic appendicectomy, in the absence of alternate pathology, should be removed [6]. The decision to proceed with removal should be with caution, ideally with a pre-operative high index of suspicion of *Enterobius vermicularis* infection, hence reducing the risk of complications, such as peritoneal contamination from appendiceal perforation [3,4,6]. This may later produce granulomata in the liver, spleen or kidney [9].

An awareness of these issues has led to the recommendation of several precautionary intra-operative steps to minimise peritoneal contamination. These include placing the specimen bag inside the abdominal cavity prior to division of the appendix, a staged division of the appendix with inspection of the lumen to facilitate retrieval of worms with endoscopic suction and thermal desiccation using endoscopic scissors with supplementary diathermy. Finally, surgeons should also carefully inspect the port site through which the appendix is retrieved, and use endoscopic suction for any spillage [6].

The identification and histopathological diagnosis of *Enterobius vermicularis* is crucial because pharmacological eradication with an anti-helminthic, such as mebendazole, is necessary to cure the basic pathologic process [4,7-9]. Pharmacological regimes consist of an initial dose followed by a second dose two weeks later. This is because the medication does not reliably kill pinworm eggs, and therefore a second dose is required to prevent reinfection by adult worms that may hatch from any eggs not killed by the first treatment [7]. Additionally, it is recommended that all household members be treated at the same time due to transmission from environmental exposure such as contaminated surfaces, clothes and bed linen, and close contact between infected patients. This also reduces the risk of re-infection [5,7,9].

Our case report shares similarities with the studies cited for patients presenting with appendicular colic secondary to *Enterobius vermicularis*. These include the typical symptoms of acute right iliac fossa abdominal pain, nausea and anorexia, with a mild-grade fever. The literature contrasts in the laboratory test results, with one large retrospective cohort study demonstrating a statistically significant normal white blood cell count and C-reactive protein [3]. Our case reflects the elevated white blood cell count that is often associated with acute appendicitis. We highlight the importance of eliciting and examining for pruritis ani, particularly in young children and those with similarly affected household relatives. A high index of suspicion should also be held for a patient with clinically diagnosed appendicitis but normal ultrasound scan, in order to lower the rate of negative appendicectomy and optimise management. Furthermore, our report is supported by a thorough literature review of the pertinent symptomatology and clinical features, investigations and peri-operative management considerations that a clinician should refer to when reviewing a patient with an atypical presentation of appendicular colic.

In summary, the literature provides compelling evidence for the importance of *Enterobius vermicularis* symptomatology awareness and thorough history taking. In particular, due diligence to perform a comprehensive judgement of risk factors for parasitic infection should be undertaken. The possibility of intestinal parasitic infection, such as *Enterobius vermicularis*, of the appendix should also be considered in the differential diagnosis of appendicular colic, especially in young women. Moreover, the incorporation of diagnostic tests should be advocated into the routine screening of appendicitis. Indeed, these were valuable lessons learnt by our surgical team.

## Conclusions

This case adds to the increasing body of literature that emphasises the importance of symptomatology awareness and maintaining a high index of suspicion for* Enterobius vermicularis* infestation in patients, particularly young females, who present with pruritus ani and appendicitis-like symptoms. Knowledge of pre-operative, intra-operative and post-operative considerations will help facilitate the ideal surgical and pharmacological management of patients, and will prevent an unnecessary appendectomy.
